# Gut *Roseburia* is a protective marker for peritoneal metastasis of gastric cancer

**DOI:** 10.1002/cam4.70037

**Published:** 2024-08-07

**Authors:** Dandan Yu, Qilin Fan, Jinru Yang, Min Jin, Linli Shi, Bin Zhou, Lei Zhao, Jieying Zhang, Zhenyu Lin, Tao Zhang, Hongli Liu

**Affiliations:** ^1^ Cancer Center, Union Hospital, Tongji Medical College Huazhong University of Science and Technology Wuhan People's Republic of China; ^2^ Institute of Radiation Oncology, Union Hospital, Tongji Medical College, Huazhong University of Science and Technology Wuhan People's Republic of China; ^3^ Hubei Key Laboratory of Precision Radiation Oncology Wuhan People's Republic of China; ^4^ Department of Gastroenterology, General Hospital of Central Theater Command Wuhan People's Republic of China

**Keywords:** 16 s rRNA sequencing, gastric cancer, gut microbiome, peritoneal metastasis, *Roseburia*

## Abstract

**Background:**

Gastric cancer (GC), particularly for advanced stage of GC, commonly undergoes peritoneal metastasis (PM), which is the leading cause of GC‐related death. However, there currently has no reliable biomarker to predict the onset of GCPM. It is well known that the imbalance of gut microbiota contributes to the development and metastasis of gastrointestinal tumors. Unfortunately, little is known about how the alternation in gut microbiota is associated with the onset of GCPM.

**Methods:**

Our current study analyzed structural characteristics and functional prediction of gut microbiota in GC patients with PM (PM group) and without PM (non‐PM group). Fresh fecal samples were collected from a discovery cohort (PM = 38, non‐PM = 54) and a validation cohort (PM = 15, non‐PM = 21) of GC patients and their 16S ribosomal RNA (16s rRNA) gene amplicons were sequenced, followed by bioinformatics.

**Results:**

The results indicated an increase in the biodiversity of gut microbiota in the non‐PM group of the discovery cohort, compared with the PM group. Moreover, LEfSe analysis found 31 significantly different microorganisms, of which the *Roseburia* ranked the fifth in the random forest (RF) model. The characteristics of intestinal microbiota in GCPM patients were changed, and the abundance of *Roseburia* in gut microbiota from the GCPM patients was reduced and receiver operating characteristic (ROC) analysis revealed that the reduced abundance of gut *Roseburia* effectively predicted the onset of GCPM.

**Conclusion:**

This signature was also observed in the validation cohort. Therefore, *Roseburia* is a protective microbial marker and the reduced abundance of *Roseburia* in gut microbiota may help early diagnosis of GCPM.

## 1 INTRODUCTION

Gastric cancer (GC) is the fifth most commonly malignant tumor, and the fourth leading cause of cancer‐related mortality in the world.^1^ About 15% to 43% of GC, particularly for those with advanced GC, undergo peritoneal metastasis (PM).^2^, ^3^ It is widely accepted that GC cells detach from the primary site through serosal invasion, subsequently adhering to the distant peritoneum, infiltrating the sub‐peritoneal space under suitable conditions, where they persistently proliferate, leading to PM.^4^ Recent advances in surgical techniques, chemotherapy, radiotherapy, and immunotherapy have improved the prognosis of GC patients.^5^ However, the overall survival (OS) rate of GC patients has not been significantly increased, and the 5‐year OS rate of GC patients after surgery remains at approximately 30%.^5^ Currently, there is no reliable biomarker to predict the early onset of PM. In clinical settings, the GCPM is diagnosed, typically based on the elevated levels of serum tumor markers, imaging examinations, or peritoneal lavage fluid cytology. However, when these indicators appear, the GC has already advanced into the late stage. Hence, it is imperative to identify convenient, non‐invasive, and cost‐effective biomarkers for predicting the development of GCPM to facilitate its early detection and treatment.

Human intestinal microbiota and their metabolites have been extensively investigated, and a recent study examines a comprehensive set of 1324 genera across integrated datasets.^6^ Intestinal flora is crucial for numerous biological processes, including metabolism, energy storage, immune responses, and intestinal functions.^7^, ^8^ In a healthy condition, intestinal microbiota can maintain the dynamic balance of their different species in the host through symbiosis, competition, and antagonism. The imbalance of gut microbiota is associated with the development of varying diseases. A recent study has shown that the diverse intracellular microbiota within the gastrointestinal tract significantly contributes to the process of tumor metastasis and colonization. This novel perspective may help in comprehending the relationship between microorganisms and tumor metastasis.^9^ It is notable that the imbalance of gut microbiota is implicated in the initiation and progression of GC. However, the precise role and mechanisms underlying the action of gut microbiota in GCPM have not been clarified. Therefore, this study aimed to investigate the relationship between gut microbiota and onset of GCPM, with the hope of gaining valuable biomarkers for predicting the early onset of GCPM.

## 2 MATERIALS AND METHODS

### 2.1 Patients

A total of 92 GC patients were recruited at the Cancer Center of Union Hospital, Tongji Medical College, Huazhong University of Science and Technology between August 2020 and January 2022. The inclusion criteria were (1) pathologically confirmed GC; (2) no history of non‐surgical anti‐tumor treatment, and their stool samples were collected 3 weeks after the surgical resection of tumors; (3) no treatment with antibiotics or other drugs that may affect intestinal flora in the week prior to samples collection; (4) no recently intestinal invasive procedure, such as gastrointestinal endoscopy and enema; (5) all subjects signed the written informed consent. The exclusion criteria were (1) people had undergone gastric cancer‐related treatments, such as surgery, chemotherapy, radiation therapy, and immunotherapy; (2) pregnant women were involved; (3) samples were from oral, skin, or oropharyngeal; and (4) changes in the gut microbiota cultured in specific media were excluded since the culture conditions exert a significant influence on microbiota data. After propensity score matching (PSM), those with statistical difference in baseline information were excluded. All 92 GC patients with metastasis were stratified into the PM (*n* = 38) and non‐PM (*n* = 54) groups, based on the evident metastatic organs.

### 2.2 Sample collection and 16S rRNA gene sequencing

Fresh stool samples (approximately 50 mg each) were collected from each subject by a trained medical stuff prior to non‐surgical anti‐tumor treatment. Each sample was carefully placed in a sterile specimen collection box and promptly transferred to a freezer of −80°C. Total DNA was extracted from individual samples using the Omega Mag‐Bind Soil DNA kit (Omega Bio‐Tek, Norcross, GA, USA), and the quality and quantity of individual DNA samples were assessed.^10^ The V3V4 regions of each qualified sample were sequenced using the Illumina platform (Illumina, San Diego, CA, USA). The original data were filtered by the dada2 using Quantitative Insights into Microbial Ecology2 (QIIME2) software (v2019.4),^11^ and the qualified data were collected and stored in FASTQ format. Subsequently, the DNA sequences were clustered as amplicon sequence variants (ASVs), and classified using the Naive Bayes classifier in QIIME2 software, followed by comparing those sequences with the data in the Greengenes database (release 13.8, http://greengenes.secondgenome.com)^12^ for species annotation.

### 2.3 Statistical and bioinformatic analyses

The categorical and continuous data were analyzed by Chi‐square test and Student's *t*‐test using SPSS software (version 22.0, SPSS Inc., Chicago, IL, USA). Statistical significance was defined when a two‐tailed *p*‐value of <0.05. The charts were generated using GraphPad Prism (version 7.0, GraphPad Software, San Diego, CA, USA). The 16S rRNA gene sequencing data were analyzed using QIIME2 software. Hypothesis testing of intergroup diversity was performed using the Kruskal–Wallis test, permutational multivariate analysis of variance (PERMANOVA), and other appropriate methods. KEGG database was used for alignments of the 16S rRNA gene sequencing data in this study.

## 3 RESULTS

### 3.1 Demographic and clinical characteristics of studying subjects

Among the 92 GC patients with metastasis, 38 (41.30%) had detectable PM, while 54 (58.70%) had non‐PM. Among those with GCPM, 52.63% of patients were > 60‐years‐old, and 63.16% were male. Their tumors were predominantly found in the gastric body (28.95%) and antrum/pylorus (31.58%). Pathological classification revealed many cases with low differentiation adenocarcinoma. In the study cohort, there was no significant difference in the demographic and clinical measures between the PM and non‐PM groups of patients (Table 1).

**TABLE 1:** Demographic characteristics of PM (*n* = 38) and non‐PM (*n* = 54) patients in discovery cohort.

Variable	PM (*n* = 38)	Non‐PM (*n* = 54)	*χ* ^2^	*p*
Age (years)				
<40	4 (10.53%)	1 (1.85%)	7.675	0.104
40–59	14 (36.84%)	24 (44.44%)		
≥60	20 (52.63%)	29 (53.7%)		
Gender				
Male	24 (63.16%)	43 (79.63%)	3.678	0.159
Female	14 (36.84%)	11 (20.37%)		
Tumor sites				
Cardiac/fundus	5 (13.16%)	23 (42.59%)	9.393	0.052
Body	11 (28.95%)	10 (18.52%)		
Antrum/pylorus	12 (31.58%)	10 (18.52%)		
Lesser/greater curvature	5 (13.16%)	6 (11.11%)		
Other	5 (13.16%)	5 (9.26%)		
Histology				
Low differentiation	23 (60.53%)	26 (48.15%)	1.373	0.241
Other	15 (39.47%)	28 (51.85%)		
Pathological types				
Adenocarcinoma	25 (65.79%)	42 (77.78%)	—	0.109
Other	0 (0.00%)	2 (3.70%)		
Signet‐ring‐cell carcinoma	13 (34.21%)	10 (18.52%)		
Whether metastasis				
Yes	38 (100.00%)	54 (100.00%)	—	—
No	0 (0.00%)	0 (0.00%)		
Metastasis sites				
Liver	0 (0.00%)	24 (44.44%)	—	—
Lung	0 (0.00%)	5 (9.26%)		
Distant lymph node	0 (0.00%)	21 (38.89%)		
Appendix	0 (0.00%)	1 (1.86%)		
Bone	0 (0.00%)	3 (5.55%)		
Peritoneum	38 (100.00%)	0 (0.00%)		
Lines of treatment				
First‐line	38 (100.00%)	54 (100.00%)	—	—
Postoperative	0 (0.00%)	0 (0.00%)		
Whether surgery				
Yes	30 (78.95%)	41 (75.93%)	0.116	0.734
No	8 (21.05%)	13 (24.07%)		

### 3.2 16S rRNA gene sequencing and sequencing data processing

**FIGURE 1:**
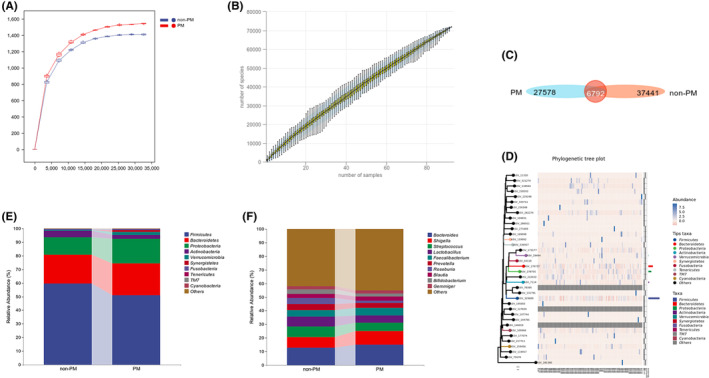
Analysis of 16 s rRNA gene sequencing data and species composition in the PM and non‐PM groups of patients in the discovery cohort. (A) Refraction curves; (B) species accumulation curves; (C) venn gram of ASV/OTUs; (D) phylogenetic tree plot; species composition of each group at the phylum level (E), and at the genus level (F).

Analysis of 16S rRNA gene sequencing data indicated that over 50,000 annotated species were identified in the fecal samples collected from this population and a similar pattern of the adequacy of sequencing data and the richness of species was observed in both the PM and non‐PM groups of patients (Figure 1A,B). The Venn diagrams displayed the number of shared and unique operational taxonomic units' (OTUs) in each group and there were 6792 shared OTUs out of 71,811 OTUs between the PM and non‐PM groups in the discovery cohort (Figure 1C). Similarly, there were 2628 shared OTUs out of 29,811 OTUs between the PM group and non‐PM groups in the validation cohort (Figure S2B).

### 3.3 Diversity analysis of intestinal microbial

**FIGURE 2:**
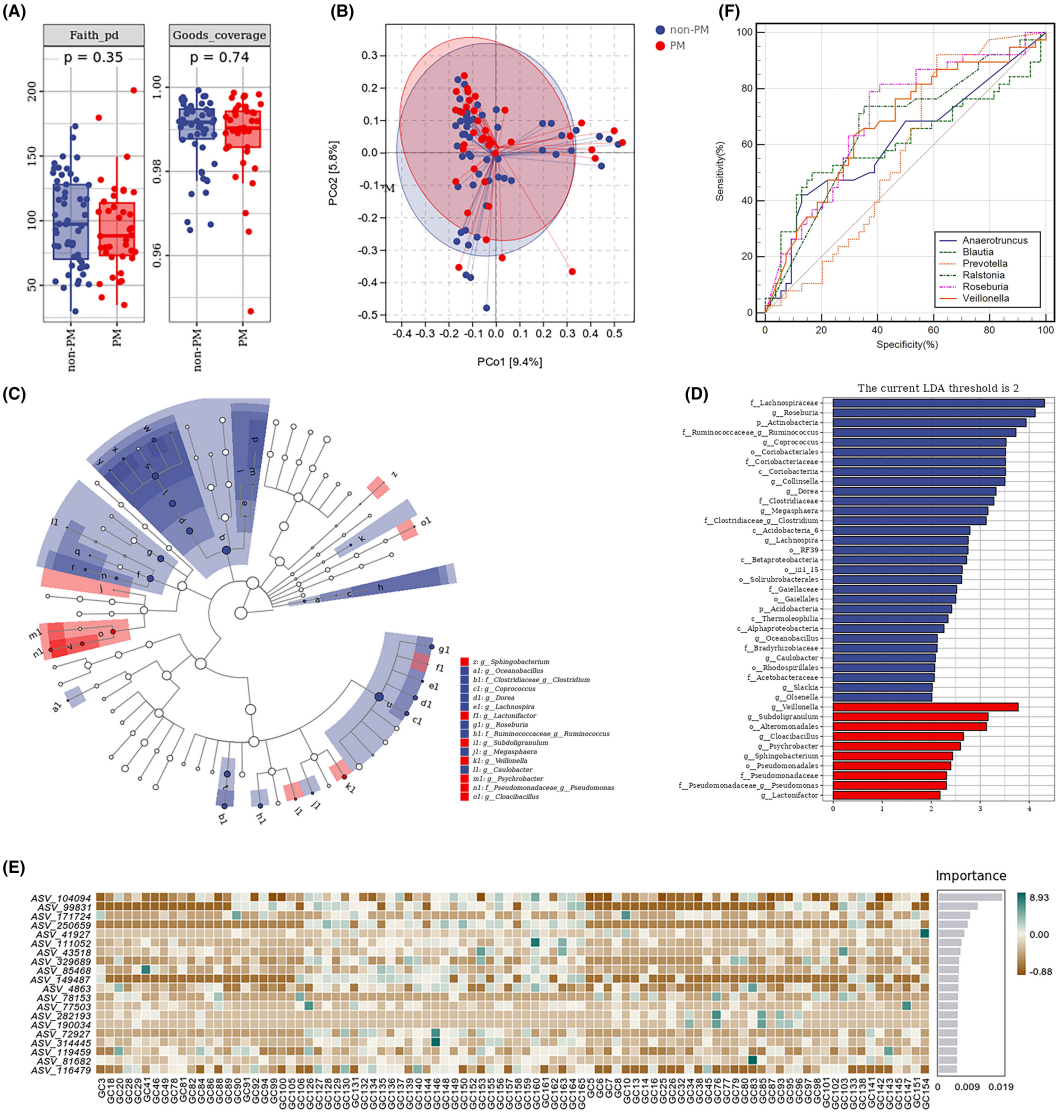
Species diversity of gut microbiota from the PM and non‐PM groups of patients in the discovery cohort. (A) Alpha diversity; (B) PCoA of beta diversity (pseudo‐F = 0.958, *p* = 0.532); (C) Taxonomic branch diagram of LEfSe (LDA threshold = 2); (D) LDA histogram; (E) RF model; (F) ROC curves.

The richness and diversity of gut microbiota in fresh fecal samples were compared between the PM and non‐PM groups of patients. There was a discrepancy in alpha‐diversity between these two groups, and the Faith_pd and Goods_coverage indexes in the non‐PM group tended to be slightly higher than that in the PM group in the discovery cohort, which was statistically insignificant (*p* = 0.35, *p* = 0.74, Figure 2A). A similar pattern of them was observed in the validation cohort (*p* = 0.31, *p* = 0.60) (Figure S1A). Principal coordinate analysis (PCoA) using the Bray–Curtis distance algorithm revealed a slight difference in beta diversity between these two groups, which was also similar in the validation cohort (Figure 2B, Figure S1B).

### 3.4 Difference species analysis between the PM and non‐PM groups

Next, the gut microbial composition between the PM and non‐PM group was examined. Initially, we examined the prevalence of various species in fecal samples at the phylum level in both the discovery and validation cohorts. Notably, four species, namely *Firmicutes*, *Bacteroidetes*, *Proteobacteria*, and *Actinobacteria*, were significantly enriched in both groups, and they ranked the top four among all microbiota in both cohorts (Figure 1E, Figure S2C).

Among the identified species, the relative abundance of *Proteobacteria* and *Bacteroidetes* in the PM group was 5.24% and 2.29% higher than that in the non‐PM group (Figure 1E). Conversely, the relative abundance of *Firmicutes* in the PM group was 8.61% less than that in the non‐PM group in the discovery cohort. At the genus level, the relative abundance of *Streptococcus*, *Prevotella*, *Lactobacillus*, and *Roseburia* in the non‐PM group was 1.73%, 0.77%, 1.81%, and 3.96% higher than that in the PM group. Notably, *Roseburia* exhibited a 2.7‐fold increase in the non‐PM group in the discovery cohort (Figure 1F). At the genus level, the relative abundance of *Bacteroides*, *Shigella*, and *Faecalibacterium* was highly enriched in the PM group (Figure S2D), whereas *Roseburia* was significantly enriched in the non‐PM group *in* the validation cohort (Figure S2D). Shigella seems to be sufficiently higher in PM group than non‐PM group at the phylum level (Figure S2).

**FIGURE 3:**
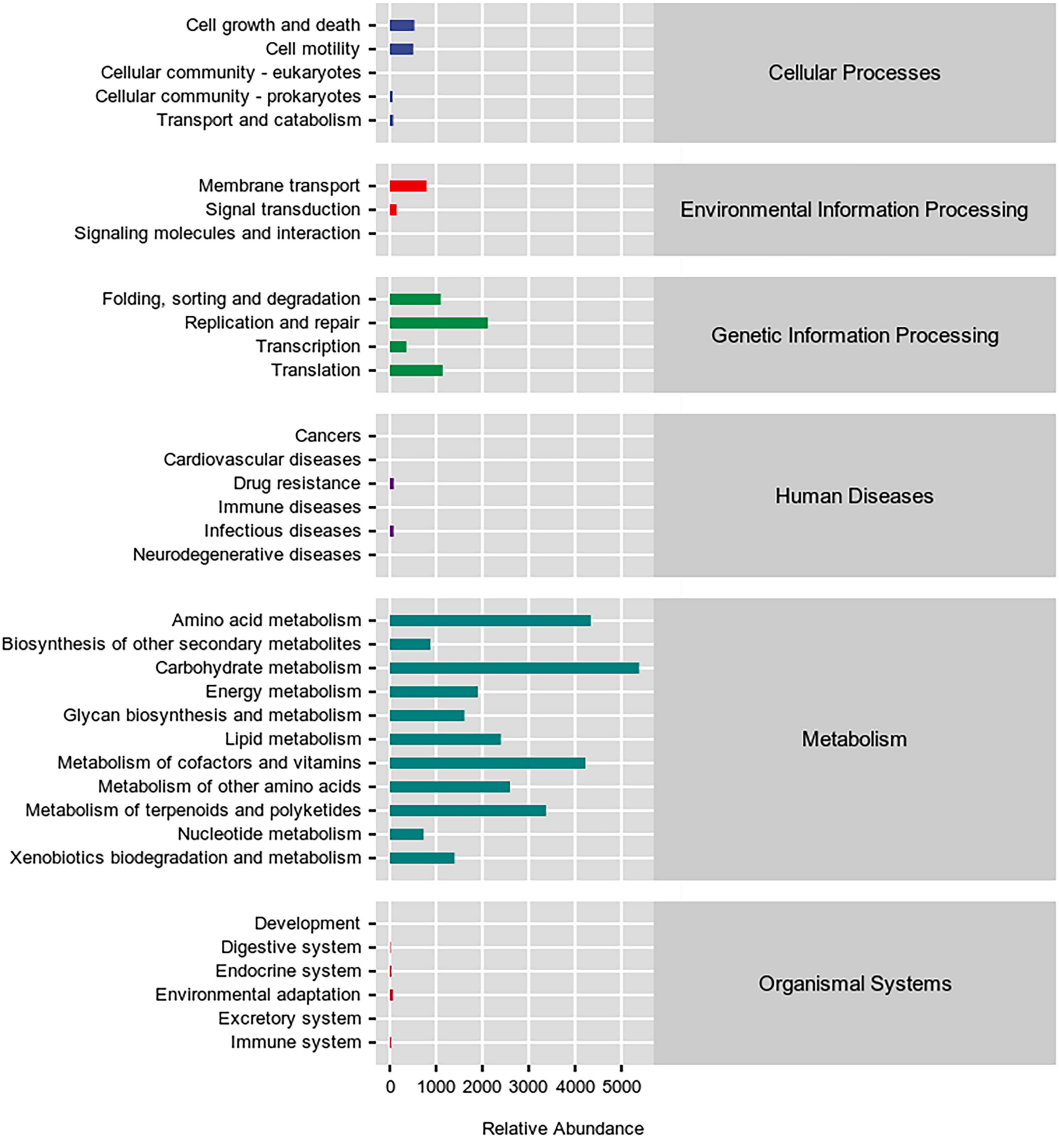
KEGG analysis of metabolic pathways in patients with GC metastasis in the discovery cohort.

Linear discriminant analysis (LDA) was used for determining the effect size (LEfSe) of each distinct flora. At a LDA of 2, and the *p* < 0.05 after correcting false discovery rate (FDR), fecal samples from the PM group of the discovery cohort exhibited 10 microorganisms that were significantly different, namely *Veillonella*, *Subdoligranulum*, *Alteromonadales*, *Cloacibacillus*, *Psychrobacter*, *Sphingobacterium*, *Pseudomonadales*, *Pseudomonadaceae*, *Pseudomonas*, and *Lactonifactor* (Figure 2C,D). In the discovery cohort, 31 significant microbes were identified in the non‐PM group, including *Lachnospiraceae*, *Roseburia*, *Actinobacteria*, and *Ruminococcus* (Figure 2C,D). Additionally, 19 significant phylotypes were identified, with 8 abundant bacteria in the non‐PM group and 11 abundant species in the PM group in the validation cohort (Figure S1C,D). Subsequently, analysis of the 16S rRNA gene sequencing data using the phylogenetic investigation of communities by reconstruction of unobserved states (PICRUSt2) software, together with Kyoto Encyclopedia of Genes and Genomes (KEGG) pathway database (http://www.genome.jp/kegg/pathway.html), annotated the functional path. The gut microbiota from GC patients with metastasis were categorized into six distinct classifications, namely cellular processes, environmental information processing, genetic information processing, human diseases, metabolism, and organismal systems (Figure 3 and Table 2). Notably, the carbohydrate metabolism pathway was significantly enriched, suggesting a potential association between metabolic disorder and GC metastasis. However, there was no significant difference in the pathways between the PM and non‐PM groups in the discovery cohort.

**TABLE 2:** KEGG functional pathway analysis in PM and non‐PM groups in discovery cohort.

Classifications	Functional pathways	Relative abundance
Metabolism (*n* = 11, average = 2620.44)	Amino acid metabolism	4338.1
	Biosynthesis of other secondary metabolites	877.05
	Carbohydrate metabolism	5383.63
	Energy metabolism	1900.9
	Glycan biosynthesis and metabolism	1610.46
	Lipid metabolism	2397.53
	Metabolism of cofactors and vitamins	4223.56
	Metabolism of other amino acids	2594.62
	Metabolism of terpenoids and polyketides	3372.32
	Nucleotide metabolism	729.26
	Xenobiotics biodegradation and metabolism	1397.35
Genetic information processing (*n* = 4, average = 1178.02)	Folding, sorting, and degradation	1099.13
	Replication and repair	2117.16
	Transcription	356.4
	Translation	1139.39
Environmental information processing (*n* = 3, average = 321.23)	Membrane transport	793.68
	Signal transduction	143
	Signaling molecules and interaction	0
Cellular processes (*n* = 5, average = 234.30)	Cell growth and death	529.60
	Cell motility	505.66
	Transport and catabolism	75.19
	Cellular community—prokaryotes	61.05
	Cellular community—eukaryotes	0
Human diseases (*n* = 6, average = 27.97)	Infectious diseases	82.24
	Neurodegenerative diseases	0.33
	Cardiovascular diseases	0.17
	Cancers	0.14
	Drug resistance	84.69
	Immune diseases	0.25
Organismal systems (*n* = 6, average = 39.30)	Environmental adaptation	68.86
	Endocrine system	32.51
	Immune system	23.78
	Digestive system	12.40
	Development	0
	Excretory system	0

### 3.5 Gut *Roseburia* is a protective marker for peritoneal metastasis of gastric cancer

The random forest (RF) analysis identified the top 20 important species. Their abundance distribution in individual samples of both cohorts is exhibited in Figure 2E, Figure S1E. The top five ASV IDs and their corresponding species in the discovery cohort included *Roseburia*, *Veillonella*, *Blautia*, *Ralstonia*, and *Anaerotruncus (*Table 3). The top five ASV IDs and their corresponding species in the validation cohort contained *Anaerotruncus*, *Blautia*, *Prevotella*, *Ralstonia*, and *Roseburia* (Table S2). LEfSe analysis of the different flora in the non‐PM group revealed that *Roseburia* was significantly distinct. Further ROC curve analysis of the predictive ability of *Roseburia* for GCPM revealed an area under the curve (AUC) of 0.698 (*p* < 0.001), indicating that a decrease in the abundance of *Roseburia* was associated with the occurrence of GCPM (Table 3 and Figure 2F). Consistently, this finding was observed in the validation cohort (Table S2 and Figure S2A). Therefore, *Roseburia* may serve as a protective microbial marker for GCPM, as its higher abundance was associated with a lower likelihood of GCPM occurrence.

**TABLE 3:** Random Forest model predicts the biomarkers for GCPM diagnosis in discovery cohort.

Order	ASV	Bacteria	AUC	SE	*p*	Enriched group by LEfSe
1	ASV_104094	*Roseburia*	0.698	0.0561	**<0.001**	Non‐PM
2	ASV_99831	*Veillonella*	0.672	0.0580	**<0.050**	PM
3	ASV_171724	*Blautia*	0.608	0.0653	0.097	—
4	ASV_250659	*Ralstonia*	0.660	0.0556	**<0.050**	—
5	ASV_41927	*Anaerotruncus*	0.609	0.0609	0.072	—

Bold indicates statistically significant.

To investigate whether reduced abundance of *Roseburia* serves as a specific indicator of GCPM, we collected stool samples from GC patients with or without metastasis (M vs. non‐M), single PM and multiple PMs (s‐PM vs. m‐PM), and PM vs. non‐PM, liver metastasis and non‐liver metastasis (LiM vs. non‐LiM), lung metastasis and non‐lung metastasis (LuM vs. non‐LuM), lymph node metastasis and non‐lymph node metastasis (LyM vs. non‐LyM), and bone metastasis and non‐bone metastasis (BM vs. non‐BM). Statistical analysis revealed that the content of *Roseburia* in the non‐M group of patients was significantly higher than that in the PM patients, but the ROC analysis unveiled that it had no prediction effect (Table 4). More importantly, the abundance of *Roseburia* was greater in the non‐PM patients than in the PM patients and the decreased abundance of *Roseburia* effectively predicted the onset of GCPM. Therefore, the higher abundance of *Roseburia*, the less likely to develop PM, while the decrease in the abundance of *Roseburia* specifically predicted the onset of GCPM.

**TABLE 4:** Comparison of *Roseburia*'s predictive role in different metastasis sites in discovery cohort.

Metastasis sites	M (Mean)	Non‐M (Mean)	M *n* (%)	Non‐M *n* (%)	*p*	AUC	SE	*p*
M versus non‐M	2245.60	2859.76	92 (50.55%)	90 (49.45%)	**<0.001**	0.508	0.0433	0.862
s‐PM versus m‐PM	669.00	1566.73	16 (42.11%)	22 (57.89%)	**0.002**	0.534	0.0966	0.497
PM	1596.86	3623.75	38 (41.30%)	54 (58.70%)	**<0.001**	0.698	0.0561	**<0.001**
LiM	3116.82	1864.43	28 (30.43%)	64 (69.57%)	**<0.001**	0.620	0.066	0.070
LuM	2355.90	2232.15	10 (10.87%)	82 (89.13%)	**<0.001**	0.514	0.107	0.896
LyM	2604.32	1604.24	59 (64.13%)	33 (35.87%)	**<0.001**	0.605	0.0608	0.085
BM	3064.71	2542.35	13 (14.13%)	79 (85.87%)	**<0.001**	0.683	0.077	0.074

Bold indicates statistically significant.

## 4 DISCUSSION

The association of gut microbiota with human health has attracted wide attention due to the proposal of the Human Microbiome Project (HMP) and advances in high‐throughput sequencing, metagenomics, biochip technology, and bioinformatics. The intestinal flora is crucial for physiological functions and health of human body. Additionally, the imbalance of gut microbiota participates in the development and progression of GC,^13^ suggesting that analysis of gut microbiota may be valuable for early detection and diagnosis of GC and PM. This study included a cohort of 182 GC patients with 50 healthy controls after calculating propensity score.^9^

In Table S1, there was a significant increase in the abundance of gut *Aquabacterium*, in GC patients, indicating its potential as a predictive biomarker for early detection of GC (Table S1). Furthermore, the abundance of gut *Roseburia* in the PM patients was significantly lower than that in the non‐PM patients (Figure 1 and Figure S2C,D). More importantly, LEfSe, RF, and ROC analyses revealed that the decrease in the abundance of gut *Roseburia* effectively predicted the onset of PM in GC patients. *Roseburia* is classified within the taxonomic ranks of *p_Firmicutes*, *c_Clostridia*, *o_Clostridiales*, and *f_Lachnospiraceae*, and is a Gram‐positive probiotic that constitutes 2.3% of the overall population of intestinal bacteria in healthy human.^14^ Moreover, *Roseburia* can produce short chain fatty acids (SCFAs), particularly butyrate.^15^ These metabolites can regulate energy generation and inflammation against pathogens.^16^ The decrease in the abundance of gut *Roseburia* has been implicated in numerous metabolic pathways and associated with the pathogenesis of various diseases, such as colorectal cancer (CRC),^17^, ^18^ inflammatory bowel disease (IBD),^19^, ^20^ irritable bowel syndrome (IBS),^21^ obesity,^22^ type 2 diabetes (T2D),^23^ neurological diseases^24^ and liver diseases.^25^ Consequently, *Roseburia* may be a valuable biomarker for early detection, and therapeutic target for treatment of these diseases.

However, little is known on the role of *Roseburia* in the etiology and progression of cancer. The current study has revealed that *Roseburia* may be a protective factor and reliable biomarker for GCPM. While many studies concentrate on the role of gut microorganisms in metastasis of CRC,^26^, ^27^, ^28^ there is a paucity of research on the context of GC.

This study includes noteworthy strengths and limitations. To the best of our knowledge, this is the first report on metabolic dysregulation in the intestinal microbiota of individuals with GC and metastasis to date. Moreover, we use uniform operating procedures to obtain uniform tissue samples. Also, we analyzed the common clinical variables in detail and comprehensively. It is worth noting that our research group has successfully established a comprehensive specimen bank comprising intestinal microbes from GC patients both prior to and post‐treatment. Our research also exists several obvious drawbacks. The propensity matching might cause the loss of information, leading to an inclusion bias. Furthermore, the food consumption of individual patients might influence their intestinal microbiota, affecting the results. Moreover, due to the ongoing treatment phase and the absence of sufficient follow‐up time, we did not include the treatment‐related data in this study.

We recognized that our analysis did not yield statistically significant difference in the pathways between the PM and non‐PM groups of GC patients. To elucidate these pathways more accurately, future investigations should employ more advanced sequencing techniques, such as single‐cell sequencing and mass flow cytometry, to discern the differential pathways between these groups of patients and investigate the underlying mechanisms. In the future, we intend to undertake comprehensive researches to elucidate the potential synergistic actions between tumor treatment and microbial modulation. It is important to note that this study does not encompass an exploration of the underlying mechanisms. Therefore, we eagerly anticipate that scholars will build upon our research findings to understand the mechanisms actions of gut microbiota in the pathogenesis, diagnosis, and treatment.

## 5 CONCLUSION

In conclusion, our data indicated that the intestinal microbial composition of patients with GCPM was different from those without metastasis and the abundance of gut *Roseburia* in GCPM patients was significantly less than those without metastasis. Therefore, the abundance of gut *Roseburia* may be a valuable biomarker for predicting the onset of GCPM.

## AUTHOR CONTRIBUTIONS

**Dandan Yu:** Conceptualization (lead); data curation (equal); formal analysis (equal); funding acquisition (equal); investigation (equal); methodology (equal); project administration (equal); resources (equal); software (equal); supervision (equal); validation (equal); visualization (equal); writing – original draft (equal); writing – review and editing (equal). **Qilin Fan:** Conceptualization (equal); data curation (equal); formal analysis (equal); investigation (equal); methodology (equal); project administration (equal); resources (equal); software (equal); supervision (equal); validation (equal); visualization (equal); writing – original draft (equal); writing – review and editing (equal). **Jinru Yang:** Conceptualization (equal); data curation (equal); formal analysis (equal); funding acquisition (equal); investigation (equal); methodology (equal); project administration (equal); resources (equal); software (equal); supervision (equal); validation (equal); visualization (equal); writing – original draft (equal); writing – review and editing (equal). **Min Jin:** Conceptualization (equal); data curation (equal); formal analysis (equal); funding acquisition (equal); investigation (equal); methodology (equal); project administration (equal); resources (equal); software (equal); supervision (equal); validation (equal); visualization (equal); writing – original draft (lead); writing – review and editing (equal). **Linli Shi:** Conceptualization (equal); data curation (equal); formal analysis (equal); funding acquisition (equal); investigation (equal); methodology (equal); project administration (equal); resources (equal); software (equal); supervision (equal); validation (equal); visualization (equal); writing – original draft (equal); writing – review and editing (equal). **Bin Zhou:** Conceptualization (equal); data curation (equal); formal analysis (equal); funding acquisition (equal); investigation (equal); methodology (equal); project administration (equal); resources (equal); software (equal); supervision (equal); validation (equal); visualization (equal); writing – original draft (equal); writing – review and editing (equal). **Lei Zhao:** Conceptualization (equal); data curation (equal); formal analysis (equal); funding acquisition (equal); investigation (equal); methodology (equal); project administration (equal); resources (equal); software (equal); supervision (equal); validation (equal); visualization (equal); writing – original draft (equal); writing – review and editing (equal). **Jieying Zhang:** Conceptualization (equal); data curation (equal); formal analysis (equal); funding acquisition (equal); investigation (equal); methodology (equal); project administration (equal); resources (equal); software (equal); supervision (equal); validation (equal); visualization (equal); writing – original draft (equal); writing – review and editing (equal). **Zhenyu Lin:** Conceptualization (equal); data curation (equal); formal analysis (equal); funding acquisition (equal); investigation (equal); methodology (equal); project administration (equal); resources (equal); software (equal); supervision (equal); validation (equal); visualization (equal); writing – original draft (equal); writing – review and editing (equal). **Tao Zhang:** Conceptualization (equal); data curation (equal); formal analysis (equal); funding acquisition (equal); investigation (equal); methodology (equal); project administration (equal); resources (equal); software (equal); supervision (equal); validation (equal); visualization (equal); writing – original draft (equal); writing – review and editing (equal). **Hongli Liu:** Conceptualization (lead); data curation (lead); formal analysis (lead); funding acquisition (lead); investigation (lead); methodology (lead); project administration (lead); resources (lead); software (lead); supervision (lead); validation (lead); visualization (lead); writing – original draft (lead); writing – review and editing (lead).

## FUNDING INFORMATION

This work was supported by the grants from the Scientific research project of Hubei Provincial Health Commission (No. WJ2023M92), the National Natural Science Foundation of China (No. 81872429), Chinese Society of Clinical Oncology Research Foundation (No. Y‐tongshu2021/ms‐0107), Beijing Bethune Public Welfare Foundation (No. BJ‐GYQZHX2021006), and Hubei Xiaoping Chen Science and Technology Development Foundation Jingrui Development Fund (No. CXPJJH122006‐1003).

## CONFLICT OF INTEREST STATEMENT

The authors declare no potential conflicts of interest.

## ETHICS STATEMENT

This study was approved by the Ethics Committee of the Union Hospital, Tongji Medical College, Huazhong University of Science and Technology (No. 2014‐041).

## Supporting information


**Figure S1.** Species diversity of gut microbiota from the PM and non‐PM groups of GC patients in the validation cohort. (A) Alpha diversity; (B) PCoA of beta diversity; (C) Taxonomic branch diagram of LEfSe (LDA threshold = 2); (D) LDA histogram; (E) RF model.

**Figure S2.** Analysis of 16 s rRNA gene sequencing data and species composition of gut microbiota from the PM and non‐PM groups of GC patients in the validation cohort. (A) ROC curves; (B) Venn gram of ASV/OTUs; Species composition of each group at the phylum level (C), and at the genus level (D); (E) phylogenetic tree plot.

Table S1.

Table S2.

## Data Availability

We have uploaded the raw data covered in this article to the SRA database (ID: PRJNA909044).

## References

[1] Röcken C . Predictive biomarkers in gastric cancer. J Cancer Res Clin Oncol. 2023;149(1):467‐481. 36260159 10.1007/s00432-022-04408-0 PMC9889517

[2] Huang X‐Z , Pang M‐J , Wang Z‐N , et al. Single‐cell sequencing of ascites fluid illustrates heterogeneity and therapy‐induced evolution during gastric cancer peritoneal metastasis. Nat Commun. 2023;14(1):822. 36788228 10.1038/s41467-023-36310-9 PMC9929081

[3] Thomassen I , Van Gestel YR , Van Ramshorst B , et al. Peritoneal carcinomatosis of gastric origin: a population‐based study on incidence, survival and risk factors. Int J Cancer. 2014;134:622‐628. 23832847 10.1002/ijc.28373

[4] Cortés‐Guiral D , Hübner M , Alyami M , et al. Primary and metastatic peritoneal surface malignancies. Nat Rev Dis Primers. 2021;7(1):91. 34916522 10.1038/s41572-021-00326-6

[5] Alsina M , Arrazubi V , Diez M , Tabernero J . Current developments in gastric cancer: from molecular profiling to treatment strategy. Nat Rev Gastroenterol Hepatol. 2023;20(3):155‐170. 36344677 10.1038/s41575-022-00703-w

[6] Ai B , Mei Y , Liang D , Dong Y , et al. Uncovering the special microbiota associated with occurrence and progression of gastric cancer by using RNA‐sequencing. Sci Rep. 2023;13(1):5722. 37029259 10.1038/s41598-023-32809-9 PMC10082026

[7] Deschasaux M , Bouter KE , Prodan A , et al. Depicting the composition of gut microbiota in a population with varied ethnic origins but shared geography. Nat Med. 2018;24(10):1526‐1531. 30150717 10.1038/s41591-018-0160-1

[8] Hartmann P , Chu H , Duan Y , Schnabl B . Gut microbiota in liver disease: too much is harmful, nothing at all is not helpful either. Am J Physiol Gastrointest Liver Physiol. 2019;316(5):G563‐G573. 30767680 10.1152/ajpgi.00370.2018 PMC6580239

[9] Fu A , Yao B , Dong T , et al. Tumor‐resident intracellular microbiota promotes metastatic colonization in breast cancer. Cell. 2022;185(8):1356‐1372.e26. 35395179 10.1016/j.cell.2022.02.027

[10] Yu D , Yang J , Jin M , et al. Fecal streptococcus alteration is associated with gastric cancer occurrence and liver metastasis. MBio. 2021;12(6):e0299421. 34872346 10.1128/mBio.02994-21 PMC8649758

[11] Rai SN , Qian C , Pan J , et al. Microbiome data analysis with applications to pre‐clinical studies using QIIME2: statistical considerations. Genes Dis. 2019;8(2):215‐223. 33997168 10.1016/j.gendis.2019.12.005 PMC8099687

[12] DeSantis TZ , Hugenholtz P , Larsen N , et al. Greengenes, a chimera‐checked 16S rRNA gene database and workbench compatible with ARB. Appl Environ Microbiol. 2006;72(7):5069‐5072. 16820507 10.1128/AEM.03006-05 PMC1489311

[13] Hunt RH , Camilleri M , Crowe SE , et al. The stomach in health and disease. Gut. 2015;64(10):1650‐1668. 26342014 10.1136/gutjnl-2014-307595 PMC4835810

[14] Hiippala K , Jouhten H , Ronkainen A , et al. The potential of gut commensals in reinforcing intestinal barrier function and alleviating inflammation. Nutrients. 2018;10(8):988. 30060606 10.3390/nu10080988 PMC6116138

[15] Tamanai‐Shacoori Z , Smida I , Bousarghin L , et al. Roseburia spp.: a marker of health? Future Microbiol. 2017;12:157‐170. 28139139 10.2217/fmb-2016-0130

[16] Nie K , Ma K , Luo W , et al. Roseburia intestinalis: a beneficial gut organism from the discoveries in genus and species. Front Cell Infect Microbiol. 2021;11:757718. 34881193 10.3389/fcimb.2021.757718 PMC8647967

[17] Liang Q , Chiu J , Chen Y , et al. Fecal bacteria act as novel biomarkers for noninvasive diagnosis of colorectal cancer. Clin Cancer Res. 2017;23(8):2061‐2070. 27697996 10.1158/1078-0432.CCR-16-1599

[18] Yu J , Feng Q , Wong SH , et al. Metagenomic analysis of faecal microbiome as a tool towards targeted non‐invasive biomarkers for colorectal cancer. Gut. 2017;66(1):70‐78. 26408641 10.1136/gutjnl-2015-309800

[19] Imhann F , Vich Vila A , Bonder MJ , et al. Interplay of host genetics and gut microbiota underlying the onset and clinical presentation of inflammatory bowel disease. Gut. 2018;67(1):108‐119. 27802154 10.1136/gutjnl-2016-312135 PMC5699972

[20] Kellermayer R . Roseburia species: prime candidates for microbial therapeutics in inflammatory bowel disease. Gastroenterology. 2019;157(4):1164‐1165. 31356805 10.1053/j.gastro.2019.05.073

[21] Chassard C , Dapoigny M , Scott KP , et al. Functional dysbiosis within the gut microbiota of patients with constipated‐irritable bowel syndrome. Aliment Pharmacol Ther. 2012;35(7):828‐838. 22315951 10.1111/j.1365-2036.2012.05007.x

[22] Haro C , Montes‐Borrego M , Rangel‐Zúñiga OA , et al. Two healthy diets modulate gut microbial community improving insulin sensitivity in a human obese population. J Clin Endocrinol Metab. 2016;101(1):233‐242. 26505825 10.1210/jc.2015-3351

[23] Qin J , Li Y , Cai Z , et al. A metagenome‐wide association study of gut microbiota in type 2 diabetes. Nature. 2012;490(7418):55‐60. 23023125 10.1038/nature11450

[24] Sampson TR , Debelius JW , Thron T , et al. Gut microbiota regulate motor deficits and neuroinflammation in a model of Parkinson's disease. Cell. 2016;167(6):1469‐1480. e12. 27912057 10.1016/j.cell.2016.11.018 PMC5718049

[25] Seo B , Jeon K , Moon S , et al. Roseburia spp. abundance associates with alcohol consumption in humans and its administration ameliorates alcoholic fatty liver in mice. Cell Host Microbe. 2020;27(1):25‐40. e6. 31866426 10.1016/j.chom.2019.11.001

[26] Wong CC , Jun Y . Gut microbiota in colorectal cancer development and therapy. Nat Rev Clin Oncol. 2023;20(7):429‐452. 37169888 10.1038/s41571-023-00766-x

[27] Dougherty MW , Jobin C . Intestinal bacteria and colorectal cancer: etiology and treatment. Gut Microbes. 2023;15(1):2185028. 36927206 10.1080/19490976.2023.2185028 PMC10026918

[28] Liu Y , Lau HC , Cheng WY , Yu J . Gut microbiome in colorectal cancer: clinical diagnosis and treatment. Genomics Proteomics Bioinformatics. 2023;21(1):84‐96. 35914737 10.1016/j.gpb.2022.07.002 PMC10372906

