# Supplementary figures and images for: Gut Roseburia is a protective marker for peritoneal metastasis of gastric cancer
Source: Cancer Med. 2024 Aug 7;13(15):e70037. doi: 10.1002/cam4.70037 (PMC11304227; doi:10.1002/cam4.70037)

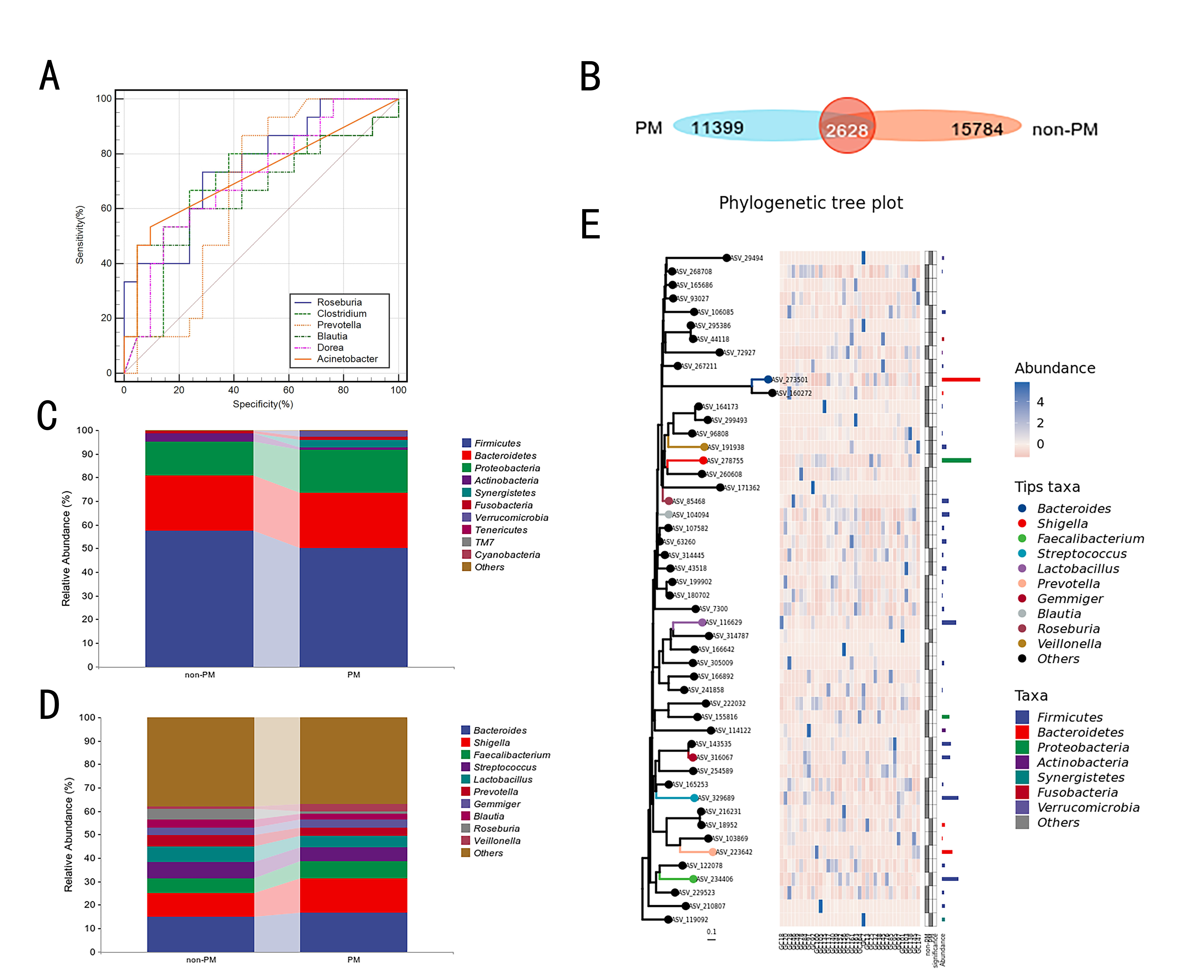

Supplement: Supplementary file 2 — Figure S2. Analysis of 16 s rRNA gene sequencing data and species composition of gut microbiota from the PM and non‐PM groups of GC patients in the validation cohort. (A) ROC curves; (B) Venn gram of ASV/OTUs; Species composition of each group at the phylum level (C), and at the genus level (D); (E) phylogenetic tree plot. [file CAM4-13-e70037-s001.jpg]
