# Supplementary material for: Gut Roseburia is a protective marker for peritoneal metastasis of gastric cancer
Source: Cancer Med. 2024 Aug 7;13(15):e70037. doi: 10.1002/cam4.70037 (PMC11304227; doi:10.1002/cam4.70037)
Supplement: Supplementary file 3 — Table S1. [file CAM4-13-e70037-s004.docx]

**Supplementary Table 1. Random Forest model predicts the biomarkers for GC diagnosis**

| Order | ASV | Bacteria | AUC | SE | *P* | Enriched group by LEfSe |
| --- | --- | --- | --- | --- | --- | --- |
| 1 | ASV_119459 | *Aquabacterium* | 0.768 | 0.0358 | **<0.001** | / |
| 2 | ASV_241713 | *Lactobacillus* | 0.833 | 0.0345 | **<0.001** | GC |
| 3 | ASV_121799 | *Ochrobactrum* | 0.848 | 0.0313 | **<0.001** | N |
| 4 | ASV_307655 | *Rubrivivax* | 0.687 | 0.0348 | **<0.001** | / |
| 5  6 | ASV_178236  ASV_66969 | *Sediminibacterium*  *Streptococcus* | 0.898  0.872 | 0.0280  0.0304 | **<0.001**  **<0.001** | /  GC |
