# Supplementary material for: Gut Roseburia is a protective marker for peritoneal metastasis of gastric cancer
Source: Cancer Med. 2024 Aug 7;13(15):e70037. doi: 10.1002/cam4.70037 (PMC11304227; doi:10.1002/cam4.70037)
Supplement: Supplementary file 4 — Table S2. [file CAM4-13-e70037-s003.docx]

**Supplementary Table 2. Random Forest model predicts the biomarkers for GCPM diagnosis in validation cohort**

| **Order** | **ASV** | **Bacteria** | **AUC** | **SE** | ***P*** | **Enriched group by LEfSe** |
| --- | --- | --- | --- | --- | --- | --- |
| 1 | ASV_41927 | *Anaerotruncus* | 0.609 | 0.0609 | 0.072 | / |
| 2 | ASV_171724 | *Blautia* | 0.608 | 0.0653 | 0.097 | / |
| 3 | ASV_111052 | *Prevotella* | 0.570 | 0.0600 | **0.024** | / |
| 4 | ASV_250659 | *Ralstonia* | 0.660 | 0.0556 | **0.004** | / |
| 5 | ASV_104094 | *Roseburia* | 0.698 | 0.0561 | **<0.001** | non-PM |
